# Seasonality, climate change, and food security during pregnancy among Indigenous and non-Indigenous women in rural Uganda: Implications for maternal-infant health

**DOI:** 10.1371/journal.pone.0247198

**Published:** 2021-03-24

**Authors:** Julia M. Bryson, Kaitlin Patterson, Lea Berrang-Ford, Shuaib Lwasa, Didacus B. Namanya, Sabastian Twesigomwe, Charity Kesande, James D. Ford, Indigenous Health Adaptation to Climate Change Research Team, Sherilee L. Harper

**Affiliations:** 1 Department of Population Medicine, University of Guelph, Guelph, Ontario, Canada; 2 Michael G. DeGroote School of Medicine, McMaster University, Hamilton, Ontario, Canada; 3 Priestley International Centre for Climate, University of Leeds, Leeds, United Kingdom; 4 Department of Geography, Geo-Informatics and Climatic Sciences, Makerere University, Kampala, Uganda; 5 Department of Community Health, Ugandan Ministry of Health, Kampala, Uganda; 6 Batwa Development Program, Buhoma, Uganda; 7 School of Public Health, University of Alberta, Edmonton, Alberta, Canada; Medical Research Council, SOUTH AFRICA

## Abstract

**Background:**

Climate change is expected to decrease food security globally. Many Indigenous communities have heightened sensitivity to climate change and food insecurity for multifactorial reasons including close relationships with the local environment and socioeconomic inequities which increase exposures and challenge adaptation to climate change. Pregnant women have additional sensitivity to food insecurity, as antenatal undernutrition is linked with poor maternal-infant health. This study examined pathways through which climate change influenced food security during pregnancy among Indigenous and non-Indigenous women in rural Uganda. Specific objectives were to characterize: 1) sensitivities to climate-associated declines in food security for pregnant Indigenous women; 2) women’s perceptions of climate impacts on food security during pregnancy; and 3) changes in food security and maternal-infant health over time, as observed by women.

**Methods:**

Using a community-based research approach, we conducted eight focus group discussions—four in Indigenous Batwa communities and four in non-Indigenous communities—in Kanungu District, Uganda, on the subject of climate and food security during pregnancy. Thirty-six women with ≥1 pregnancy participated. Data were analysed using a constant comparative method and thematic analysis.

**Results:**

Women indicated that food insecurity was common during pregnancy and had a bidirectional relationship with antenatal health issues. Food security was thought to be decreasing due to weather changes including extended droughts and unpredictable seasons harming agriculture. Women linked food insecurity with declines in maternal-infant health over time, despite improved antenatal healthcare. While all communities described food security struggles, the challenges Indigenous women identified and described were more severe.

**Conclusions:**

Programs promoting women’s adaptive capacity to climate change are required to improve food security for pregnant women and maternal-infant health. These interventions are particularly needed in Indigenous communities, which often face underlying health inequities. However, resiliency among mothers was strong and, with supports, they can reduce food security challenges in a changing climate.

## Introduction

Climate change is projected to have substantial impacts on global food security, the effects of which are already being felt [1–4]. Food security can be defined as having sufficient available food which is stable in supply, nutritious, and accessible to individuals and households (FAO, 2008). Changes in temperature, precipitation, and extreme weather patterns that influence crop production and yields, agricultural pests, and diseases, are some of the biggest threats to food security [3, 5, 6]. This is a particular challenge for countries in sub-Saharan Africa, where food security is already a major health issue [5, 7–12]. Globally, sub-Saharan Africa has the highest prevalence of undernourishment at over 22% of the population [1]; climate change-associated declines in food security can hamper efforts to reduce malnutrition, thus propagating poor health outcomes [13]. Consequently, it is important to understand how climate change will affect human nutrition and health via food security.

Women in low-resource areas are at higher risk of negative health impacts due to climate change [14–17]. Pregnant women are especially sensitive because of pregnancy-associated morbidities and specific health needs [16, 18]. Food insecurity and undernutrition during pregnancy is associated with maternal micronutrient deficiencies [13, 19], depression and anxiety [20–22], gestational diabetes and hypertension [23], and mortality [13]. For many women in low-resource areas, achieving proper nutrition during pregnancy is difficult due to insufficient food availability and access, especially during the hunger seasons when food is scarcest (typically the dry season) [18, 24–27], and this struggle is likely to be intensified by climate change-associated declines in food security [1–4]. While we know that climate and meteorological conditions can impact food security, an understanding of the pathways and mechanisms by which these influences are exerted in the antenatal period is not well-defined.

Food security in a changing climate is also a growing concern for many Indigenous peoples [9, 10, 28–31]. Traditional foods ranging from wild fruits to hunted game have been negatively impacted by climate change for diverse populations, from Inuit [30] and First Nations [31] communities in Canada to the Shawi and Shipibo in Peru [29]. A high dependence on the land, discrimination, and lack of representation in decision-making groups, among other factors, result in increased challenges and restricted adaptive capacity for Indigenous communities in a changing climate—including the impacts on food security [9, 29, 32–34]. In Uganda, the Indigenous Batwa have experienced significant disparities in areas including health, education, and income following forced eviction from their traditional lands [9], all of which influence their ability to access food.

The projected declines in food security associated with climate change and the risks posed to mothers and infants in sub-Saharan Africa, especially in Indigenous communities, are complex, challenging, and under-researched. Some quantitative projects have investigated seasonality of nutrient intake during pregnancy [24–27, 35], but little research has documented the pathways through which climate causes variation in food security and nutrition for this population. As such, this study explored maternal food security, nutrition, and antenatal health among rural Ugandan women in the context of climate change. Specific objectives were to characterize: 1) sensitivities to climate-associated declines in food security for pregnant Indigenous women; 2) women’s perceptions of climate impacts on food security during pregnancy; and 3) changes in food security and maternal-infant health over time, as observed by women. A greater understanding of these relationships will be important for informing future health interventions and promoting maternal-infant wellbeing in a changing climate.

## Methods

### Conceptual framework & approach to research

**Fig 1:**
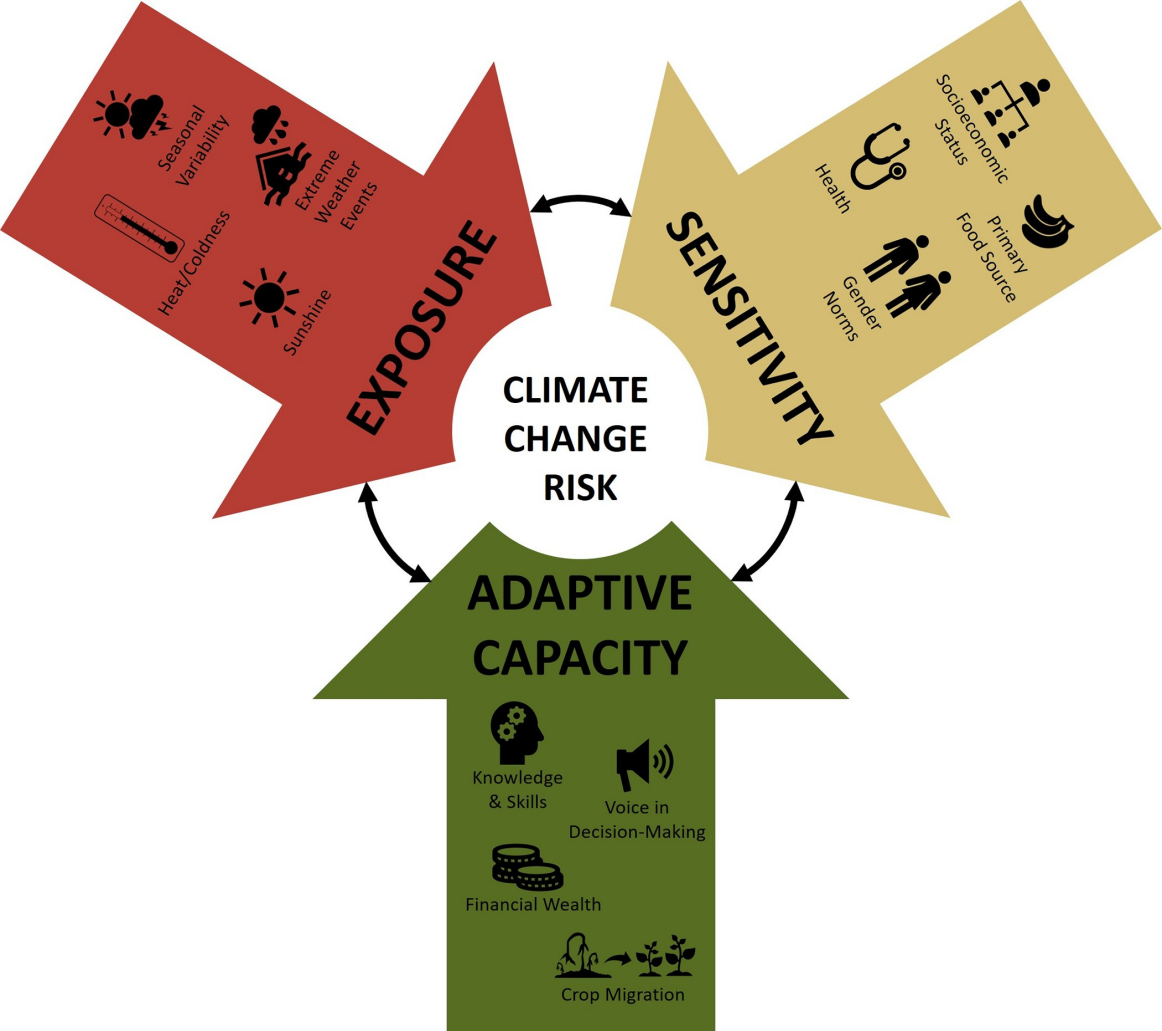
Conceptual framework highlighting factors affecting climate change risk in rural Uganda.

This study was rooted in a climate change conceptual framework developed by Ford et al. [33] and adapted for use in the Ugandan context as described by Berrang-Ford et al. [9]. This framework characterizes susceptibility to harm as a function of exposure and sensitivity to the impacts of climate change, and one’s adaptive capacity to manage negative outcomes and leverage changes in climate as opportunities and benefits when possible [9] (Fig 1). For the purposes of this study, exposure is defined as any climate effects capable of impacting maternal food security and health, while sensitivity describes the factors which influence the degree to which these exposures impact the mother. Adaptive capacity refers to the differing abilities of individuals, communities, and health systems to respond to climate change exposures based on resources including tangible assets, knowledge, and decision-making power. The framework acknowledges that while many people can have similar climate change exposures, the severity of their impact and the ability of individuals and communities to mitigate exposures is highly variable.

A community-based research approach was utilized [36, 37], whereby local partners with whom the research team has built relationships (since 2009) were engaged in every step of the research process from defining the research question to validation of findings. In 2016, while developing a research grant proposal in partnership with communities, community members identified food security and maternal health as key research priorities. After working with communities to define the research project, we further engaged the Batwa Development Program, a local organization working with the Batwa, and Bwindi Community Hospital to understand how findings could be used to improve their services. We then worked with women from the Batwa communities, along with community leaders, to develop research methods and manage project administration. Finally, our research team consisted of Indigenous community members, as well as local health practitioners who were selected by the communities. This study was conducted according to the guidelines laid down in the Declaration of Helsinki and all procedures involving research study participants were approved by the University of Guelph Research Ethics Board (REF #16MY016), Makerere University School of Social Sciences Research Ethics (REF #MAKSS REC 04 17 044), and the Uganda National Council for Science and Technology (REF #SS 4334). Written or verbal informed consent was obtained from all subjects/patients. Verbal consent was witnessed and formally recorded.

### Community partners

Approximately 252,000 people live in Kanungu District in southwest Uganda [38], the majority belonging to the Bakiga ethnic group, a group who do not self-identify as Indigenous, and who are a traditionally agrarian society. Approximately 700 Indigenous Batwa live in the District [9]. The Batwa are regarded as among the first inhabitants of the Great Lakes region in central Africa and were the only people living in the forests of southwestern Uganda until the mid-16^th^ century [39, 40]. They were forest hunter-gatherers until their forced eviction without compensation from Bwindi Impenetrable Forest National Park in the 1990s to create a wildlife reserve [39]. They were given little land and housing, and alternative livelihood options were not provided, resulting in limited access to capital and sustainable income [40]. Discrimination and low education rates continue to limit adaptive capacity and restrict the Batwa’s ability to influence policy at a systemic level, although they petitioned the Constitutional Court of Uganda in 2013 to recognize and compensate them for the historical and ongoing injustices they have faced [40]. The case is ongoing as of 2020. Dispossession from their traditional lands and the subsequent transition to an unfamiliar agrarian lifestyle has contributed to negative health outcomes in the Batwa community, including higher rates of acute gastrointestinal illness [41] and malaria [42, 43] compared to local and/or national averages. These health concerns also affect the Bakiga, to a lesser degree [11]. Major food sources for the Batwa and Bakiga include subsistence agriculture and manual labour in exchange for food [10, 11]. Additionally, some Batwa earn income to buy food by working in tourism [10], while some Bakiga earn income from cash crops [11]. Only 3% of Batwa households have been identified as food secure, which represents one of the lowest food security rates in the published literature [10]. The consequences of food insecurity have been significant; the Batwa have met criteria for ‘Critical health situation crisis’ designation for malnutrition by the WHO [44]. Additionally, the Batwa are highly vulnerable to climate change, which may increase the burden of disease and exacerbate chronic food security challenges in their communities [9, 10]. The Bakiga have also identified food security as an important issue; however, the prevalence of malnutrition is lower and they have more coping mechanisms to manage food shortages compared to the Batwa [11, 44].

### Data collection

**Fig 2:**
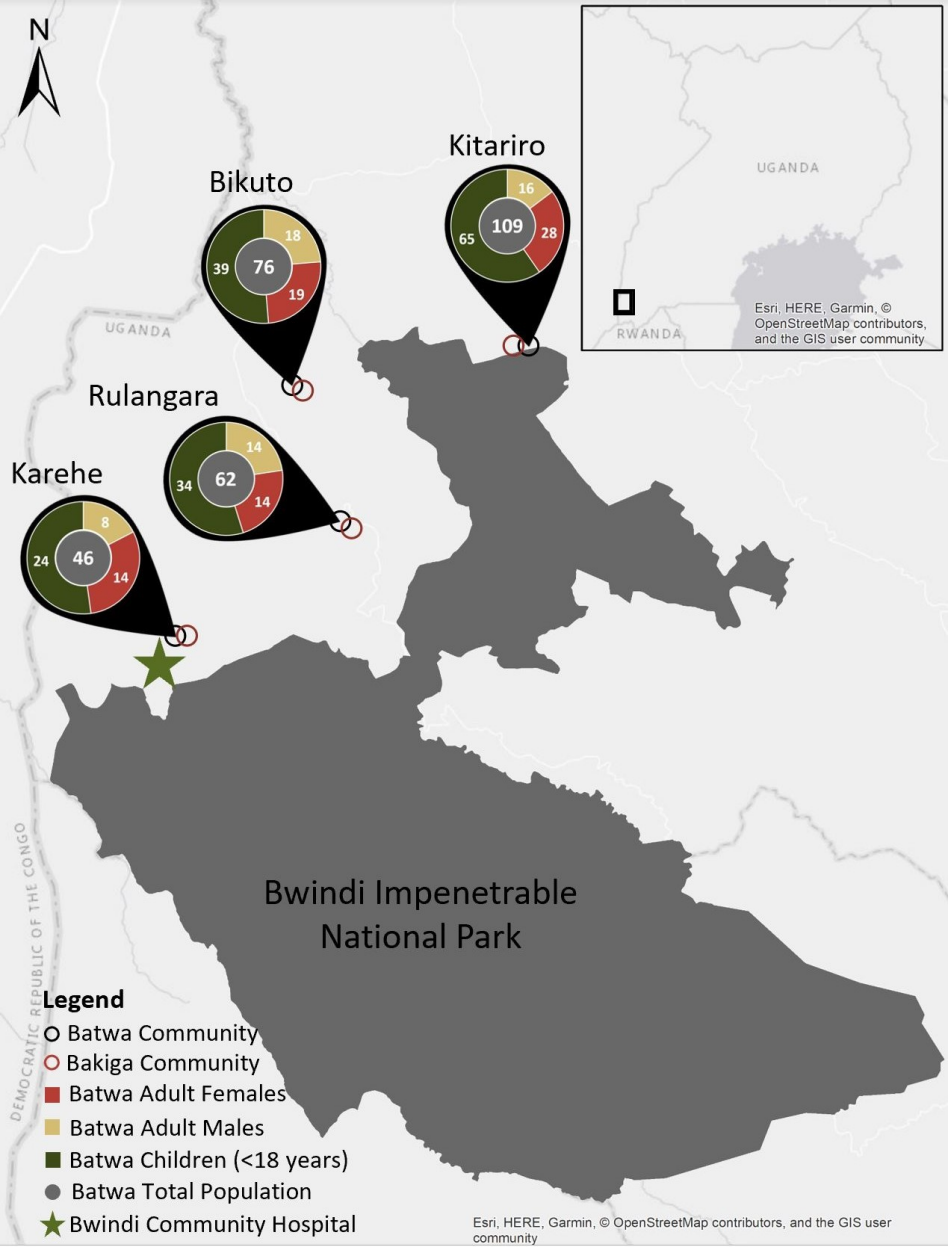
Map of community locations and Batwa populations in Kanungu District, Uganda, which participated in focus group discussions. Map not to scale. Base map and data from OpenStreetMap and OpenStreetMap Foundation. Population data for year 2017.

Focus group discussions (FGDs) were conducted in four Indigenous Batwa communities and four geographically matched Bakiga communities in the Kanungu District of Uganda (Fig 2) in May and June 2017, involving a total of 24 Batwa and 22 Bakiga women (S1 Table) ranging in age from 18 years old to elderly. FGDs allowed for in-depth exploration of nuances and complexity of lived experiences, generating rich data that more standardized questionnaires often fail to grasp. Additionally, FGDs encouraged discourse among participants that can highlight commonalities and differences in their perspectives. Local chairpersons were approached for invitations to work with communities and they facilitated contacting women and supported data collection. Communities were selected to represent a range of experiences, including varying distances from Bwindi Community Hospital and Bwindi Impenetrable National Park, which can affect health via access to care and food security via differences in land fertility and microclimates near the forest. Focus group participants were selected by convenience sampling among a census of Batwa women and a geographically matched sample of women living in the closest Bakiga community. All adult women having at least one previous or current pregnancy were eligible to participate in the discussions. Between 5–6 women were included per group to balance hearing a variety of perspectives with having enough time to explore each participant’s voice in depth. Each group involved a diversity of ages to gather an understanding of the experiences of women in different life stages and to be able to compare current and past experiences with food security during pregnancy. All FGDs were conducted in English (author J.M.B.) with simultaneous translation to the local language (Rukiga) by an experienced local researcher (author S.T.), who obtained informed consent from participants. Rukiga is spoken by both the Batwa and Bakiga. A semi-structured interview guide (S1 File) was utilized, covering topics including pregnancy diet, perceived impacts of climate/weather on nutrition and pregnancy outcomes, and observed changes in nutrition and pregnancy outcomes over time. During FGDs, the term “climate change” was not used as this nomenclature is unfamiliar to many of the participants; however, in order to capture information in the context of climate change, we focused on “long-term patterns and changes” in lived experiences with food security in the wet and dry seasons and how these have changed over time. All discussions were audio recorded with permission from participants. Focus group length averaged 52 minutes (range 44–61) and 412 total minutes of conversation were recorded.

### Data analysis

A qualitative thematic analysis of the data using a constant comparative method was conducted [45], involving the following major steps: data familiarization, generating initial codes, searching for themes, reviewing themes, and defining and naming themes. The verbatim English components of FGDs were fully transcribed and checked for accuracy against audio recordings. As the Rukiga components of the FGDs were translated in real-time to English, the Rukiga audio was not further analyzed. Transcripts were uploaded into NVivo© 10 to facilitate manual coding. Both theory-driven and data-driven codes were generated (S2 Table) [46]. Theory-driven codes were derived from the Ford et al. climate change framework (Fig 1) [9, 33] and the Food and Agriculture Organization of the United Nations dimensions of food security [47]. These codes were used to identify and classify features of interest in the data set, which were categorized and collated to identify broad patterns and themes linking climate and food security during pregnancy. Duplicate coding was not utilized, although three authors (J.M.B, K.P., & S.L.H.) reviewed the complete codebook and sections of coded FGDs together. Throughout the data collection and analysis stages, peer debriefing [48] was conducted with local experts and researchers who have worked with the communities, via both informal frequent consultations and organized seminars. The validity of the analysis was improved as these interactions allowed for triangulation of information from multiple sources [48]. An audit trail was also kept, which included all recordings and transcripts of all meetings with communities and partners, notes taken during FGDs, a log of data interpretation activities and reflections, and records of key decisions made during the data analysis process [48].

## Results

**Fig 3:**
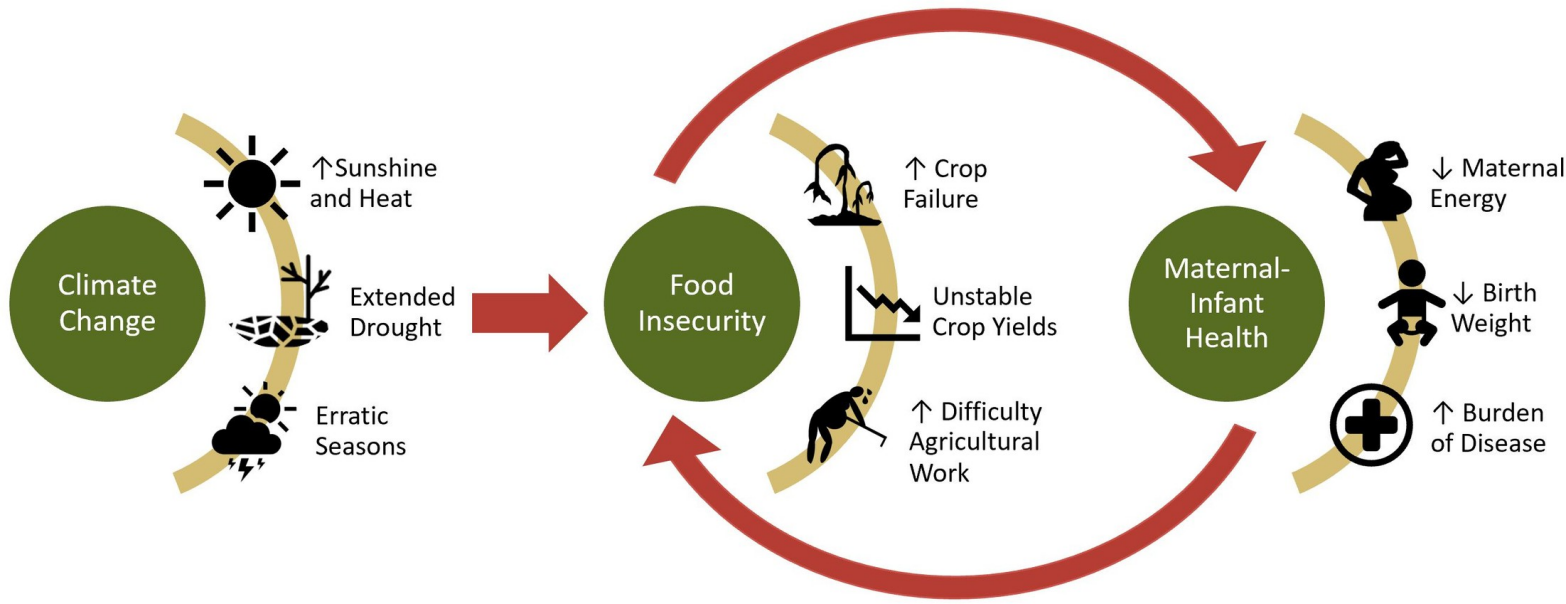
Pathways of climate change, food insecurity, and maternal-infant health reported by mothers in rural Uganda.

Women from all communities described extensive connections between seasonality, food security, and maternal health (Fig 3). Most women agreed that long-term changes in seasons and weather patterns have negatively impacted food security. Four key themes were identified: i) seasonality and climate as key modulators of food security, ii) Indigenous identity as a determinant of food insecurity severity and adaptive capacity, iii) climate exposure-sensitivity as a mediator of maternal health and food security, and iv) maternal food security as a climate-sensitive determinant of infant health.

### Seasonality and climate change as key modulators of food security

Food security was reported to differ between the dry and rainy seasons. Many women found it easier to get food in the rainy season because *“every crop grows*.*”* Multiple communities discussed the need to save food grown in the rainy season for future:

*During the dry season, it’s suffering. If you don’t save what [food] you had during the rainy season*, *then in the dry season you find you have nothing when you are pregnant*.

Some also favoured the rainy season because of the diversity in diet, saying *“You have every type of food in the rainy season…But during the dry season you find we are depending on only one or two types of food*.*”* Shorter dry periods were important for harvesting and safely drying crops. Most communities acknowledged that there are positive and negative aspects about each season for food security.

In addition to subsistence agriculture, season also impacted the ability to access paid work, for example harvesting cash crops. For women who laboured to buy food, *“During the rainy season is when we can go work outside [for others]*. *Then we can have money to buy fish and meat and rice*.*”* However, significant rain can pose problems because “*You can’t go to the garden to work when it’s raining… and you find you can’t get food*.*”* In one community, the Batwa women participated in tourism, and reported that the dry season has higher tourist volumes: *“So*, *when we get a lot of visitors*, *we earn more money and we get many types of food we want*.*”*

The amount of food intake was described as variable throughout pregnancy and generally greatest late in pregnancy, making food insecurity more detrimental to health at this time. Several women expressed interest in timing their pregnancies around the seasons when their food security is higher, but explained that there is a lack of capacity to accomplish this goal. As one Bakiga woman explained,

*I have had six pregnancies*. *I have tried to have some pregnancies be during the dry season, but others I have failed*…*I am always failing because of family planning [challenges]*.

Women from every community whose primary food source was subsistence agriculture noticed changes in weather and climate which have impacted food security while pregnant. Women identified that *“It’s harder now for us to get food when we are pregnant*, *compared to the past*, *because the seasons are changing*.” They further explained “*Now we are experiencing a lot of sunshine and drought*, *so that’s why our crops can’t grow*,*”* and *“When there is a lot of drought*, *our crops dry and we don’t have enough food…we are experiencing a lot of drought compared to the past*.*”* The unpredictability of weather was also a significant issue for ensuring a sufficient food supply: “*You find now when it should be rainy season you are experiencing a lot of sunshine*, *and during the dry season you find you experience rain*.*”* Increased plant disease and pests were also reported to decrease crop yields. Most communities expressed that these decreases in food security over time have had impacts on pregnancy, saying *“In the past we used to have food…but now people are getting pregnant and they are suffering because they don’t have food to eat during their pregnancy*.*”* Economic stressors were described as adding to the climate-related exposures, with women highlighting that *“The world is becoming harder to live in because you go and work for a full day and they pay 5000 [shillings]*, *and yet 5000 can only buy you a kilo of posho [staple cornmeal]*, *which cannot feed your family*.*”*

The participants attested that pregnant women were healthier when food security was better and the climate was stable:

*“In the past, women would be strong compared to now when they are pregnant… They had a variety of food*. *At that time, the crops they would grow, they would grow well because their land was fertile at that time compared to now. The seasons—there were not changes of the seasons like now.”*

### Indigenous identity as a determinant of food insecurity severity and adaptive capacity

Although Batwa and Bakiga women faced similar environmental exposures, Batwa women described more severe experiences than Bakiga women when discussing the dry season, noting that *“There is suffering*,*” “We just boil and drink water only*,*”* and *“Nothing is good in the dry season*.*”* Some Bakiga women mentioned having comparable experiences when they were too ill to work for food during pregnancy, but they did not typically portray these as regular occurrences. Rather, in Bakiga communities, women described their adaptive capacity to cope with the effects of unpredictable weather on agriculture:

*You may think that it’s approaching rainy season, then you go and grow crops. Then it continues with sunshine and those crops can’t germinate*… *So that’s when we try to use the swampy areas*—*some land which can retain water even in the dry season*.

No Batwa women discussed any adaptive options for agricultural activities when asked how they cope during difficult seasons, including access to swampy lands in the dry season. Rather, the Batwa described their minimal land access and ownership as a barrier to food security, stating *“Because we have little land*, *the land we use to grow*, *to cultivate [food]*, *is now overcultivated*. *So*, *we can’t grow any crops in it*,*”* and *“If we were given enough land to grow our crops*, *we would hope to have food throughout our pregnancy*.*”* A Batwa Elder also lamented their loss of access to traditional lands, where she described they used to gather honey, yams, meat and medicinal herbs which gave mothers and babies *“strong immunity*”.

The exception to the trend of Indigenous women experiencing worse food insecurity than non-Indigenous women was one community of Batwa women where most food was purchased using income from working in tourism. These women were able to afford more nutrient rich foods during pregnancy: “*We usually have enough money during the peak season of tourists*. *When we go and dance for them…we always have money to buy meat*.*”* These women also did not note differences in the seasons or weather over time affecting food security. They were the only community to indicate that food security had improved over time, which they attributed to increased tourism.

### Climate exposure-sensitivity as a mediator of maternal health and food security

Physical labour, such as working the land to grow and harvest crops, was often required of Batwa and Bakiga women to acquire food. During pregnancy, women reported experiencing negative physical health symptoms, including “*dizziness*,” “*general malaise*,” “*nausea*,*”* “*shivering*,” and feeling “*weak*,” among others. These ailments made it more difficult to work and reduced their access to food. For example, one Batwa woman explained:

*When we are pregnant, we are always facing the problem of not getting enough food*… *we don’t have enough power–energy–to go and labour for food and still we have to go because we have to eat*.

A particularly difficult pregnancy “*can make you so sick that you will not be able to work for the entire pregnancy*, *so you don’t have food*.*”* The impact of physical health symptoms associated with pregnancy was reported to vary as the pregnancy progressed, contributing to an instability of food access. According to one Batwa woman, *“In the early pregnancy we still have energy to go and work for food*, *but in late pregnancy we don’t…so*, *it’s very hard for us to get food*.” The social support women received when unable to obtain fulfill their role as primary food provider varied, with some stating “*When we are sick the men have to take over*,” while others reported they “*don’t get help at all*” from their husbands or community.

There was much discussion on how climate and meteorological conditions can influence maternal health and therefore exert an impact on food security. Women reported that pregnancy decreased their ability to manage negative health outcomes associated with climate such as fatigue, dizziness, and chills, noting that,

*When you are pregnant and you are facing these [health] challenges, you have no choice but to relax. But when you’re not pregnant*—*normally whether it’s cold, whether it is hot—you have to go out and work*.

Some women reported feeling sicker in the dry season while pregnant, noting that the heat and sunshine can lead to symptoms such as dizziness, general unwellness, and feeling uncomfortably hot. Food insecurity in the dry season also impacts health, as a Bakiga woman explained, *“In the dry season we don’t even have energy because we are not getting enough food to eat*, *so we don’t have the power to stay in the sunshine digging*.*”* However, others felt healthier during the dry season, reporting *“During the rainy season*, *a lot of diseases come up and there is a lot of coldness compared to the dry season*.*”* Frequently there was a difference of opinion within a community about which season was associated with better maternal physical health during pregnancy. However, there was consensus that having enough food makes it easier for them to handle the environmental exposures, saying *“We would be healthy if we had enough food*. *[Even if it is] raining too much or sunshine or drought…with food in the house we would be good*.*”*

### Maternal food security as a climate-sensitive determinant of infant health

Several women acknowledged better access to healthcare is improving the health of young children in recent years. Some women believed season played a role in infant health, saying *“Babies born in the rainy season*, *they are born strong because their mother usually has food to eat*.*”* Being able to access enough food throughout pregnancy was consistently identified as a factor that influenced the health of babies upon delivery: “*If you got food throughout your pregnancy you may have a healthy baby*. *If you didn’t get enough food throughout your pregnancy*, *maybe you will have an unhealthy baby*.*”* Many communities believed changes in infant health over time were linked to food security and climate. Infants born more recently were often described as *“weak*,*” “small*,*”* and *“having more sickness*.*”* One woman said, *“It’s all about the food…the mothers used to have enough food*,*”* when reasoning why infants born in the past were healthier. They further explained *“Because of the change in weather being experienced with drought*, *we don’t have enough food”* and *“The mothers do not eat*. *When we’re not having supper and lunch*, *how do you expect our kids to be born bigger*?”.

## Discussion

This study described how seasonality and climate change impact food security among pregnant women in rural southwestern Uganda, a population with unique sensitivities to food insecurity. The relationship between food security and seasonality has been well-established [10, 49–51]; however, a stronger body of qualitative research–that gives voice to women’s experiences–describing the pathways sustaining this relationship is needed, to which our work contributes. Our analysis revealed that food security had strong influences on maternal health during pregnancy, effects which were impacted by weather and becoming intensified over time by changes in local climate as described by women, with ramifications for maternal-infant health. This finding is well supported by the literature, which provides evidence that climate change is impacting maternal health through multiple pathways, including food security [52–54]. Like our participants, women in rural Burkina Faso have highlighted the consequences of food insecurity for maternal health, explaining *“‘A pregnancy during the hunger season is like a sickness…If you haven’t eaten*, *you don’t have the energy to birth a baby*,*’”* [22], a sentiment echoed by Kenyan women [55]. The widespread nature of the issue highlights its gravity and the importance of our study results, which provide insight into both pathways of vulnerability and adaptation to improve maternal food security in the context of climate change.

The most significant food security challenges women described facing were decreased food availability secondary to weather conditions limiting agricultural yields, and decreased food access secondary to physical limitations to working during pregnancy. Their attestations of longer and hotter dry seasons and increasingly unpredictable weather patterns are consistent with observed climate data and future projections for the region [5, 56, 57]. Food availability from crops is likely to continue to be a challenge under difficult growing conditions. Even if food is available, accessibility remains a barrier during pregnancy. Women reported that they bear the greatest responsibility for food acquisition in both Batwa and Bakiga communities, a task often accomplished by strenuous labour [18]. In Uganda, gender disparities in agriculture are marked; indeed, 77% of women are involved in agriculture, yet most do not own or control the land they work, women tend to have access to less advanced farming technologies, and they perform more unpaid work compared to men [58]. Climate change can exacerbate these inequities and add further burdens, such as women having to walk farther to collect water for the family during drought, in addition to their time spent working the land [58]. Similar to our results, in rural China, Mexico, and Tanzania [59] and Burkina Faso [22], women do not tend to decrease their physical labour during pregnancy, though physically intensive work is associated with negative outcomes such as maternal hypertension, miscarriage and pre-term birth [60]. Many women, like the Ugandan mothers in this study, cannot decrease their activity as a main food provider [22], and assistance from men was not guaranteed. Further, we found the interaction between physical health and food security was cyclic; illness during pregnancy decreased food procurement, and consequent undernutrition intensified illness, making it even harder for women to access food [22]. Climate change is projected to increase the frequency of extreme weather events such as heat waves and heavy precipitation [5], heightening health risks for pregnant women who must work outside for food and making it easier to enter and harder to exit the cycle of ill health and poor food security.

Indigenous Batwa women described a heightened sensitivity to climate-related decreases in food security compared to neighbouring Bakiga women. This disparity is consistent with research globally, which has found that Indigenous communities face a greater burden of negative health outcomes due to climate change than others [9, 16, 29, 34]. Although women from both Batwa and Bakiga communities discussed facing hunger during pregnancy, Batwa women discussed severe food shortages as a more regular occurrence, which is consistent with the higher rates of malnutrition [44]. Indigenous Batwa women attributed their limited adaptive capacity in this area with their relative lack of land access and ownership, factors which have been identified as strong predictors of health in their communities [9, 10]. Thus, our results suggest that the Batwa may be disproportionately challenged to deal with increasing instability in local agriculture due to climate change. The issues of land ownership and access, agricultural experience, as well as loss of Batwa traditional Indigenous nutritious food sources, all stem from the Batwa’s forced eviction from their ancestral lands without equitable compensation. Land dispossession is a common experience among many Indigenous peoples globally that negatively impacts health and limits climate change adaptive capacity [10, 61, 62]. Although not directly discussed in the FGDs, the Batwa not having equitable representation at decision-making tables, as evidenced by their eviction and subsequent decades of land access struggles despite their calls for change, is another factor dampening their ability to adapt. Thus, secure, equitable, and just land access will be critical to ensuring food security for the Batwa and other Indigenous peoples [63], especially as climate change continues to exacerbate existing health inequities.

An important exception to the disparity in food security sensitivity and adaptive capacity between Batwa and Bakiga women involved the cultural tourism industry as an alternate source of income. Unlike women working in agriculture, women who primarily worked in tourism did not note changes in weather and they reported greater food security over time; this may indicate that tourism could act as a source of adaptive capacity for these women, making them less susceptible to negative impacts of climate variability compared to women in agriculture. Climate projections of longer dry seasons in the area [5, 64] may be beneficial as this time was reported as the high season for tourist activities and the time of greatest food security for women in the industry. However, tourism is a highly location-dependant and saturable market in Bwindi [9]. Other alternative income-generating activities may be beneficial, though, as diverse income bases have more room for adaptive capacity and sustainability in the face of climate change [65]. Financial poverty is a major driver of food insecurity and limits adaptive capacity [7, 66, 67], as women cannot purchase sufficient food to supplement their family’s diet if their crop yields are low or if they cannot work due to illness or weather. Even when women have income, participants described their pay as insufficient to meet their food needs, which may be impacted by gender pay gaps [58]. Cash employment and selling of traditional handicrafts have been identified as important adaptive strategies for a flood-prone agrarian Indigenous community in Peru [68], and programs have already started that help Ugandan farmers with beekeeping, making crafts, and running small businesses to cultivate adaptive capacity to climate change [64]; similar investments could be made in Kanungu District to improve maternal food security.

Meteorological exposures, including heat, sunshine, and coldness, were often identified by Batwa and Bakiga women as influencers of maternal food security and health. Dry periods and extreme precipitation events are expected to increase regionally [5, 56, 64, 69, 70]. Intense environmental exposures such as extreme heat can be a health burden for any person [14, 71], but women in our study highlighted that, during pregnancy, exposures with which they are normally able to cope become an almost insurmountable barrier to carrying out daily functions including accessing food; this supports that pregnant women have increased sensitivity to experiencing negative health impacts of climate change [5]. Batwa and Bakiga women agreed that having sufficient food would improve their coping abilities for challenging climatic and meteorological conditions during pregnancy. A further potential adaptive strategy would be to intentionally time pregnancies such that periods of lower food intake in the pregnancy align with seasons of lower food availability, which has been proposed in the rural Indian context [27]. However, timing pregnancies is difficult due to physiological unpredictability, desirability, and local relevance, appropriateness, and context. Research in Burkina Faso has demonstrated that women do not want to get pregnant during the hungry season, but access to contraceptives and their use remains a barrier [22], much like the experiences described in our study. As well, women are not always in control of their own family planning decisions [72–74], reducing feasibility for some.

In addition to maternal health changes, many mothers noticed a decrease in health for their infants over time, a trend to which they believe climate-related undernutrition during pregnancy contributed. These observations correlate with a previous study in Kanungu District, which found that temperature and precipitation during pregnancy impacted birth weight, with an increased effect size for Batwa populations [18]. It would be reasonable to expect mothers to perceive infant health to be overall improving thanks to increased access to antenatal healthcare delivered by Bwindi Community Hospital and its satellite clinics. And yet it appears these efforts to enhance healthcare may only be buffering the impacts of deteriorating food security and malnutrition on local maternal-infant health. Globally, child undernutrition—including intrauterine growth restriction, which can be caused by antenatal undernutrition [75]—is responsible for 35% of under-five child mortality [76]; improving antenatal food security and nutrition in Uganda could have important impacts on reducing childhood deaths.

This study has some limitations. First, the concepts of food security and health are multidimensional; therefore, it can be difficult to gain an understanding of the experiences of several women in a FGD. There is potential for FGDs to be overtaken by a few dominant voices or for a collective voice to emerge which does not adequately capture divergent perspectives and experiences [77]. To mitigate these issues, strategies such as giving each woman a rock to use as a vote for key questions, such as which season is harder to obtain food, and if it is becoming easier or harder to have sufficient food over time, allowed for discourse around differing opinions and the opportunity to explore all participants’ views, which often were not unanimous. Further, the women’s comfort disclosing their experiences could have been affected by the pre-existing dynamics between group members including age and social position. However, food security and health in pregnancy are not particularly controversial in these communities as most people are facing similar challenges. As well, the positionality of some of the researchers may have influenced results. Members of the research team who were not from Uganda were reflective in considering how their positionality could influence the research process. Efforts were made to reduce the power imbalance between the FGD facilitators and participants, for example by conducting the FGDs in the communities where women live and feel comfortable, by holding discussions in groups rather than with individual women, and by having a person well-known to most of the women in his role as a researcher and healthcare provider over several years assist with translation of the FGDs. However, there is still a possibility that the asymmetry in the relationship affected the participants’ responses. Finally, data collection relied on participant recall of previous experiences with pregnancy, maternal-infant health, food security, and climate. Memory recall is subject to inaccuracy, which could have influenced discussions comparing present experiences and those from many years in the past. Including women from multiple generations in the same FGD allowed for people to challenge each others’ perceptions and conclusions about changes over time, increasing the reliability of the information. A strength of the study that helped to address all of these study limitations was the community-led design, which recognized the participants as experts in their own experiences and, as such, partners in the research process. Importantly, the study is centred around highlighting the lived experiences of rural women and Indigenous women, whose perspectives have historically been undervalued.

## Conclusion

This study provided evidence for suggested causal pathways and mechanisms through which climate change is impacting food security during pregnancy among rural Ugandan women, information which is important to informing future health interventions that work to build adaptive capacity and increase resilience to climate change in sensitive populations. The results presented here show that, for many women in rural Uganda, they consider consistent availability and access to nutritional food to be the *most important* determinant of health both during and after pregnancy. Yet, adequate food security is not being attained in a changing climate, and mothers and infants are thought to be experiencing poor health outcomes as a result. Batwa women in particular have more severe and frequent experiences with lack of food during pregnancy, largely attributable to inequities they have faced as Indigenous peoples—inequities whose impacts have been amplified for segments of the population with unique health needs like pregnant women and infants. Interventions which cultivate resiliency to climate change, such as equitable access to fertile land and alternate income generation activities, have potential to help pregnant women manage the direct effects of environmental exposures on their food security and health. These initiatives would be especially influential for building adaptive capacity among the Indigenous Batwa, and they merit further investigation. Though climate change poses a serious threat to food security, and thus maternal-infant wellbeing, women are resilient, and with proper support and partnerships they will be better able to surmount these challenges.

## Supporting information

S1 Table Participants per focus group and community populations. (DOCX)

S2 Table Thematic analysis codes and definitions. (DOCX)

S1 File Semi-structured focus group discussion interview guide. (DOCX)
