# Correction: Seasonality, climate change, and food security during pregnancy among Indigenous and non-Indigenous women in rural Uganda: Implications for maternal-infant health

**DOI:** 10.1371/journal.pone.0303592

**Published:** 2024-05-08

**Authors:** The PLOS ONE Staff

## Notice of Republication

This article was republished on April 25, 2024, to correct errors in title capitalization that were introduced during the typesetting process. The publisher apologizes for the errors. Please download this article again to view the correct version. The originally published, uncorrected article and the republished, corrected articles are provided here for reference.

## Supporting information

S1 File Originally published, uncorrected article. (PDF)

S2 File Republished, corrected article. (PDF)
