# Supplementary material for: Seasonality, climate change, and food security during pregnancy among Indigenous and non-Indigenous women in rural Uganda: Implications for maternal-infant health
Source: PLoS One. 2021 Mar 24;16(3):e0247198. doi: 10.1371/journal.pone.0247198 (PMC7990176; doi:10.1371/journal.pone.0247198)
Supplement: S1 Table — (DOCX) [file pone.0247198.s001.docx]

**S1 Table. Participants per focus group and community populations**

| **Community** | **Focus Group Discussion Participants** | **Adult Women Population (2017)** |
| --- | --- | --- |
| Kitariro (Batwa) | 6 | 28 |
| Kitariro (Bakiga) | 6 | Unknown |
| Bikuto (Batwa) | 6 | 19 |
| Bikuto (Bakiga) | 6 | Unknown |
| Rulangara (Batwa) | 6 | 14 |
| Kishanda (Bakiga) | 5 | Unknown |
| Karehe (Batwa) | 6 | 14 |
| Mukono (Bakiga) | 5 | Unknown |
