# Supplementary material for: Seasonality, climate change, and food security during pregnancy among Indigenous and non-Indigenous women in rural Uganda: Implications for maternal-infant health
Source: PLoS One. 2021 Mar 24;16(3):e0247198. doi: 10.1371/journal.pone.0247198 (PMC7990176; doi:10.1371/journal.pone.0247198)
Supplement: S2 Table — (DOCX) [file pone.0247198.s002.docx]

**S2 Table. Thematic analysis codes and definitions**

| Code | # References  (# FGDs) | Definition | Example |
| --- | --- | --- | --- |
| *THEORY-DRIVEN CODES* | | | |
| Exposure | 54 (8) | Subjection to factors in the environment which could pose a hazard to wellbeing | *Because the change of weather being experienced with drought so we don’t have enough food* |
| Sensitivity | 64 (8) | Reference to the varying severity of how people are affected by climate exposures (e.g. differences based on food source, pregnancy) | *We are saying it depends on how the pregnancy is. Sometimes it can make you so sick that you will not be able to work for the entire pregnancy, so you don’t have food.* |
| Adaptive Capacity | 14 (4) | Changing behaviour to mitigate the challenges associated with climate exposures | *We are saying to maybe to have enough food during the dry season like people have swampy areas…swampy areas where they should always prefer growing their crops in those swampy areas because they don’t usually get dried easier in the dry season.* |
| Food Security | 189 (8) | Reference to the availability, accessibility, and/or stability of food | *She is talking of gardens. They don’t yield, they don’t grow in dry season.* |
| Food availability | 48 (8) | Reference to factors which affect the ability of the food supply to meet the needs of the community (e.g. crop failure) | *During the dry season, that greens or even gardens, they dry off…so no food in the dry season.* |
| Food accessibility | 37 (8) | Reference to ability to obtain food based on personal resources like income, ability to work | *We are saying it depends on how the pregnancy is. Sometimes it can make you so sick that you will not be able to work for the entire pregnancy, so you don’t have food.* |

| Food stability | 35 (8) | Reference to temporal fluctuations in the ability to obtain food, due to factors such as health, weather, conflict | *So in early pregnancy we still have energy to go and work for food, but in late pregnancy we find we don’t have power to go and work for food. So it’s very hard for us to get food.* |
| --- | --- | --- | --- |
| *DATA-DRIVEN CODES* | | | |
| Abnormal foods | 7 (5) | Foods not normally consumed by people | *Sometimes when we are pregnant we feel that we want to take like low mango…unripe mango* |
| Agriculture | 86 (8) | Any mention of planting, growing, harvesting food. Reference to crops, gardens. | *Majority of the foods mentioned we grow them for ourselves.* |
| Alcohol | 3 (2) | Consumption of or cravings for alcohol | *I am not taking alcohol but when I am pregnant I would think of drinking alcohol.* |
| Appetite | 13 (6) | Reference to appetite for food | *In the early pregnancy we feel that we don’t have appetite for food, but when it is close to five months…that’s when we have appetite for food and we eat a lot.* |
| Batwa culture | 12 (3) | Reference to traditional Batwa lands, medicines, beliefs, language, or other cultural aspects | *We think that babies who were born in the past…they were energetic compared to those born now, because those who were born in the past used to take like local herbs, called “nah-chee-baz-ee”, it was from the forest.* |
| Changes in society | 2 (2) | Reference to changes in the way people behave, on a large community scale. | *Because we fry a lot, recently. People fry a lot and in the past we never used to fry.* |
| Climate change | 15 (8) | Changes in weather, climate and/or meteorological patterns over time | *The seasons are changing…we find we are experiencing droughts even…it’s the rainy season but you find even dry season…so drought is being prolonged compared to the past.* |
| Cold weather | 3 (2) | Cold environmental temperature | *But when you’re not pregnant, normally whether it’s cold, whether it is hot, you have to go out and work.* |
| Coldness | 8 (5) | Reference to personally feeling cold | *In the rainy season we always have coldness and we have some headache.* |
| Coping | 37 (8) | Methods of dealing with challenges | *Sometimes when we don’t have food we find we are eating some foods which we never want…which we don’t like eating…or we just boil water and drink water only if we don’t have food.* |
| Cravings | 23 (8) | Desire to eat certain foods, or non-nutritive substances | *When we are pregnant we like eating a lot of meat. We crave meat most.* |
| Crops/  Gardens | 80 (8) | Specific mention of the term crop(s) or garden(s), where food is grown | *In the rainy season, the crops grow.* |
| Crop/garden ownership | 7 (5) | Possession of crops or gardens | *We have both. We have both crops and gardens.* |
| Crop Failure | 25 (8) | Reference to crop destruction or reduction in yield due to adverse weather, pests, or other factors. | *Even too much rain can destroy our crops.* |
| Crop yields | 8 (5) | Crop productivity and yields | *Way back it was easier before than now because we would dig and the productivity was still high, like the yields were still high, but now the yields are low.* |
| Diet | 41 (8) | The kinds of foods being eaten | *Meat, fish…those are the special foods we eat when we’re pregnant.* |
| Diet diversity | 15 (6) | Reference to the variety in the types of foods being eaten | *But during the dry season you find we are depending on only one or two types of food throughout.* |
| Drought | 6 (4) | Extended period of extreme dryness and lack of rain | *So we are talking because the change of weather being experienced with drought so we don’t have enough food…so the weather goes with food.* |
| Dry season | 94 (8) | Reference to the dry season | *During the dry season it’s the harvesting time, so it’s when we have food in our stores.* |
| Eating frequency | 3 (2) | How often women are eating | *So that when we are pregnant we usually take little food in amount, but frequently. We find we eat like five times a day.* |
| Eating well | 5 (3) | Specific use of the phrase “eating well” or a close variant (to convey having enough nutritious food) | *In the past, the babies would be born strong and big, because their mothers were feeding well, and there were few diseases compared to now.* |
| Energy and strength | 32 (7) | Reference to physical power and vitality | *In the past, women would be strong compared to now when we are pregnant.* |
| Family | 11 (6) | Children, husband, extended family members | *So that when we’re pregnant we have to share with children, but the children have to get the big share.* |
| Feeling hot | 8 (5) | Reference to personally being overheated | *Because of the sunshine I am always feeling very hot all of the time.* |
| Food | 267 (8) | Any reference to food, such as food consumed during pregnancy or food grown | *In the rainy season, the crops grow…and we get vegetables…and we have enough food when we are pregnant.* |
| Food intake amount | 29 (7) | Reference to the amount of food eaten | *When we are pregnant, we take less in amount compared to when we are not pregnant.* |
| Food preferences | 8 (6) | Reference to desiring certain foods more than others | *We have food, but sometimes maybe if you feel that you want a certain type of food and you can’t afford getting it at the moment, you can be stressed for that.* |
| Food purchasing | 17 (8) | Reference to buying food for consumption | *And we are like we can even sell some cash crops like tea and coffee, then we get money to go and buy food from the shops.* |
| Food stores | 6 (4) | Reference to storing or saving food for future consumption | *During the dry season it’s suffering. But if you don’t…save…like what you had during the rainy season, then in the dry season you find you have nothing when you are pregnant.* |
| Food vs weather | 6 (5) | Comparison of the impacts of weather and food on an outcome (e.g. health, size baby) | *Yeah we would be healthy if we had enough food…raining too much or sunshine or drought, but with food in the house we would be good.* |
| Forest | 8 (2) | Reference to former home of the Batwa | *In the past we used to have like free foods from the park, from the forest.* |
| Grains | 31 (8) | Reference to any grains or grain-based foods (e.g. millet, posho, rice) | *When we are pregnant we usually prefer meat, rice, and matoke. And those are good foods.* |
| Hail | 6 (5) | Hail | *She talked of rain stones and if the rain comes with the wind you find some crops like matoke are blown off.* |
| Harvest | 10 (5) | Harvesting food from crops/gardens | *That when we plant during the rainy season, when the dry season comes we have harvested and we have dried so we have a lot of food as well.* |

| Healthcare | 16 (7) | Any care provided for physical or mental wellbeing | *So the babies of today are healthy because they are born in the hospital and they get immunization.* |
| --- | --- | --- | --- |
| Healthy food | 2 (2) | Discussion of foods identified as “healthy” or “good for you” or a similar variant | *We are told when we eat those foods we produce healthy babies and the mother is always healthy when she’s eating those ones.* |
| Heat | 4 (2) | Reference to hot environmental temperatures | *In the dry season, because of a lot of sunshine and a lot of heat, so you find maybe we are sick more because of these.* |
| Hunger | 6 (4) | Reference to hunger due to insufficient food | *And sometimes because we don’t have food with us we sleep hungry* |
| Infant health | 41 (8) | Wellbeing of infants/babies | *We are saying even the babies born in the past would be more healthy compared to the babies born now.* |
| Infection & disease | 18 (8) | Reference to general disease, specific infections (e.g. malaria, measles), and fever | *The babies born in the dry season, they usually incur a lot of diseases* |
| Inter-pregnancy variability | 3 (3) | Reference to differences in experience between pregnancies | *There are some certain times when we don’t have food in their pregnancy.*  ***And does that usually happen for every pregnancy, or not all the time?***  *No…not every pregnancy* |
| Land fertility | 6 (5) | Discussion of fertility or overcultivation of land | *We are not getting enough food compared to the past because there is overcultivation of land, so the land is now less fertile compared to the past.* |
| Land ownership | 4 (3) | Possession of land | *If we are given enough land to grow our crops we would hope to have food throughout our pregnancy because we would be having big gardens.* |
| Maternal mortality | 1 (1) | Death during pregnancy or shortly after birth | *You be scared that maybe you might die with the pregnancy* |
| Maternal health | 98 (8) | Wellbeing of women during pregnancy and shortly after birth | *In the past, the mothers would be healthy compared to now* |
| Meat & fish | 27 (8) | Any reference to meat and/or fish | *So that when we are not pregnant even we can take a month without eating fish or meat.* |
| Mental health | 22 (6) | Reference to emotions, thoughts, feelings | *We are depressed and we are stressed, because when we are pregnant you find we don’t have energy to go and work and get what we want.* |
| Money | 21 (8) | Reference to money or cost of items/services | *We can even sell some cash crops like tea and coffee, then we get money to go and buy food from the shops.* |
| Muzungus | 5 (2) | White people | *But for muzungus, for whites, for tourists, they usually give us money…in the dry season we get money for getting foods* |
| Nausea & vomiting | 7 (5) | Specific reference to feeling nauseated and/or vomiting | *We get full so fast and like we get nausea, we get nauseated, so we cannot eat a lot.* |
| Negative outcome | 105 (8) | Anything having a detrimental impact on outcomes such as health and food security | *If the rain comes with the wind you find some crops like matoke are blown off.* |
| Past vs. present | 55 (8) | Differences between past and current experiences and observations | *Even the babies born in the past would be more healthy compared to the babies born now.* |
| Pests | 5 (3) | Reference to insects, animals, or other pests affecting crops | *There has been like infestation like crop infestation, like pests and diseases have risen.* |
| Physical health | 78 (8) | Physical wellbeing, including illness, disease, energy, strength | *The babies born in the dry season they usually incur a lot of diseases.* |
| Pica | 18 (8) | Eating non-nutritive substances such as soil or stones | *Sometimes when I am pregnant I crave for soil* |
| Plant disease | 3 (2) | Reference to diseases affecting crops | *And almost every type of plant is getting a disease.* |
| Positive outcome | 38 (8) | Anything having a beneficial impact on outcomes such as health and food security | *The good part of the dry season…time for harvesting. So all our crops get dried off to be ready for storage.* |
| Powerlessness | 7 (4) | Inability to control a situation and mitigate challenges | *You only get water. At times you will be wanting to get porridge, but you have nowhere to get it from so you just suffer with your water.* |
| Pregnancy planning | 8 (6) | Attempting to control the time of pregnancy | *We don’t plan for our pregnancies according to the seasons* |
| Pregnant vs not | 21 (8) | Reference to difference in experiences when pregnancy compared to normal | *So we usually take those foods even when we are not pregnant but when we are pregnant we have appetite for those foods so we take them more* |
| Premature delivery | 2 (2) | Baby is born before pregnancy reaches full term | *Or you produce the baby before term…because you don’t have food and you’re stressed.* |
| Provider role | 5 (4) | Working to ensure family has food, schooling, and other essentials | *The world is becoming harder to live in because you go and work for a full day and they pay 5000, and yet 5000 can only buy you a kilo of posho, which cannot feed your family.* |
| Rain | 15 (7) | Reference to rain specifically (not rainy season in general) | *Yeah at times the rain is good but it comes with like hail stones…like wind with high intensity.* |
| Rainy season | 93 (8) | Reference to the rainy season specifically | *So she is talking of in the rainy season, the crops grow…and we get vegetables…and we have enough food when we are pregnant.* |
| Renting Land | 2 (2) | Renting land | *We go and hire land in the swampy areas, then we grow crops.* |
| Seasonality | 191 (8) | Reference to the seasons in general, rainy or dry seasons | *In the rainy season, the crops grow…and we get vegetables…and we have enough food when we are pregnant.* |
| Selling food | 2 (2) | Selling food for money | *We can even sell some cash crops like tea and coffee, then we get money to go and buy food from the shops.* |
| Size baby | 13 (6) | Size of baby upon delivery | *They are strong and big, those that are born in the rainy season.* |
| Social support | 7 (5) | Aid given by family, friends, or community members | *So that’s when we try to use the swampy areas…some land which can retain water even in the rai— in the dry season. So we try to share that land so as to grow crops and find food.* |
| Subsistence agriculture | 8 (4) | Reference to growing food for consumption by self or family | *Majority of the foods mentioned we grow them for ourselves.* |
| Sugary and fried foods | 4 (2) | Reference to foods containing processed sugar and/or foods fried in oil | *It’s not a problem that they are eating those fried foods…but they like them because of the way they taste.* |
| Sunshine | 21 (7) | Reference to sunshine/heat from sun | *Because of the sunshine I am always feeling very hot all of the time.* |
| Swampy Lands | 6 (3) | Using swampy or wet land to grow food | *Okay there are some people who have land in the swampy areas, so even they can be having food in the dry season because when they grow crops there, then it’s easier to grow in the dry or rainy season.* |
| Time within pregnancy | 18 (8) | Reference to different months, trimesters, or other time markers for a pregnancy | *So in the early pregnancies, you find even though we have food we don’t have appetite for…for eating* |
| Tourism | 10 (1) | Reference to tourism in the area | *So that when we have a lot of tourists it’s when we…it’s when we have money to buy all those foods.* |
| Traditional medicines | 13 (3) | Consumption of traditional herbs for medicinal purposes | *No that doesn’t happen, having special foods like herbs, we don’t.* |
| Travel | 3 (3) | Going a distance from home | *Sometimes it affects us because if we are sick so we have to be away from our home so that means we are not working and that affects us in terms of getting food.* |
| Undernutrition | 1 (1) | Explicit use of the term malnutrition or a variant, or “bad nutrition” | *But now these babies who are born nowadays they have malnutrition, they have big stomachs.* |
| Understanding eating behaviours | 9 (6) | Having an understanding (or not) of why certain foods are desired/eaten during pregnancy | ***Does anyone know why you like these things…the soil or the uncooked millet flour?***  *We don’t know.* |
| Vegetables, fruit & greens | 53 (8) | Reference to vegetables, fruits, or greens | *In the rainy season, the crops grow…and we get vegetables…and we have enough food when we are pregnant.* |
| Weather | 44 (8) | Reference to meteorological conditions or weather events | *Too much rain even can destroy crops.* |
| Wind | 5 (4) | Wind | *Yeah at times the rain is good but it comes with like hail stones…like wind with high intensity.* |
| Within-group differences | 18 (7) | Difference in experience or perception between women in the same focus group | *During the dry season she is okay compared to the rainy season. But the others are saying in the rainy season we feel okay.* |
| Work | 51 (7) | Any type of labour | *We always have power to go and work during the rainy season compared to the dry season.* |
| Work for others | 6 (2) | Labouring for other people in return for food or money | *During the rainy season is when we can be having a lot of work outside, from the Bakiga.* |
| Work for self | 2 (2) | Labouring for self-run operations (agriculture, tourism) | *That we don’t usually consider the season when we are digging because we know the season, so we usually start preparing our gardens during the dry season, then by the time when it approaches the rainy season you find the gardens are already prepared. Then we have to put the seedlings into the gardens. But, we do work hard during the rainy season.* |
