# Supplementary material for: Seasonality, climate change, and food security during pregnancy among Indigenous and non-Indigenous women in rural Uganda: Implications for maternal-infant health
Source: PLoS One. 2021 Mar 24;16(3):e0247198. doi: 10.1371/journal.pone.0247198 (PMC7990176; doi:10.1371/journal.pone.0247198)
Supplement: S1 File — (DOCX) [file pone.0247198.s003.docx]

**S1 File. Semi-structured focus group discussion interview guide**

**Topic 1: Maternal dietary patterns during pregnancy**

- Can you tell me about what kinds of foods you eat when you are pregnant?
- Is there anything special that you eat during pregnancy? If yes, why do you eat those things?
- Do you eat a different amount of food during pregnancy compared to when you are not pregnant? Does your food intake change at different times in the pregnancy?
- When does food matter most during pregnancy? Why?

**Topic 2: Food security and season**

- Who thinks it is easier to get the food you need for pregnancy in the dry season? Who thinks it is easier to get the food you need for pregnancy in the rainy season? *(Demonstrated by each woman placing a stone in a specified area to ‘vote’ for the dry vs rainy season)*
- What time of year makes for better maternal nutrition? Why is it easier? Are there any bad things about this season for getting the food you need?
- What time of year makes for worse maternal nutrition? Why is it harder? Are there any good things about this season for getting the food you need? What would make this season easier?
- When you don’t have food, what strategies do you use to make up for the lack of food? Do these strategies change when you are pregnant?

**Topic 3: Food security and maternal-infant health over time**

- Do you find that over time it is getting easier or harder to get the food you need for pregnancy? What has caused these changes?
- How does nutrition affect the health of mothers?
- How does nutrition affect pregnancy outcomes (e.g. maternal infections, miscarriage, birth weight, newborn health)?
- Do you think mothers are healthier or sicker now than they were in the past? What has caused the changes?
- Do you think babies are born healthier or sicker now than they were in the past? What has caused the changes?
